# Supplementary material for: Experimental and Bioinformatic Insights into the Effects of Epileptogenic Variants on the Function and Trafficking of the GABA Transporter GAT-1
Source: Int J Mol Sci. 2023 Jan 4;24(2):955. doi: 10.3390/ijms24020955 (PMC9862756; doi:10.3390/ijms24020955)
Supplement: Supplementary file 1 [file ijms-24-00955-s001.zip › Supplementary material.pdf]

Supplementary material

The effect of epileptogenic mutations on the function and trafficking of the GABA transporter GAT-1

Dolores Piniella[1,2], Ania Canseco[1], Silvia Vidal[3,4], Clara Xiol[3,4], Aránzazu Diaz de Bustamante[5], Itxaso Martí, Judith Armstrong[4,6.7], Ugo Bastolla[1], Francisco Zafra[1,2]

[1]Centro de Biología Molecular Severo Ochoa, Facultad de Ciencias, Consejo Superior de Investigaciones Científicas, Universidad Autónoma de Madrid, Madrid, Spain.

[2]IdiPAZ, Institute of Health Carlos III (ISCIII).

[3]Sant Joan de Déu Research Foundation, 08950 Barcelona, Spain.

[4]IPER, Institut de Recerca Pediàtrica Hospital Sant Joan de Déu, 08950 Barcelona, Spain

[5]Genetics Unit, Hospital Universitario de Móstoles, 28935 Madrid, Spain.

[6]Molecular and Genetics Medicine Section, Hospital Sant Joan de Déu, Barcelona, Spain.

[7]CIBER-ER (Biomedical Network Research Center for Rare Diseases), Institute of Health Carlos III (ISCIII), Madrid, Spain

Pediatric Neurology, Hospital Universitario Donostia, Biodonostia, Universidad del País Vasco UPV-EHU, San Sebastián, Spain.

Figure S1. Subcellular distribution of GAT-1 variants in transfected cells. COS7 cells were co-transfected with expression vectors for HA-GAT-1 WT or the indicated HA-tagged mutants- plus mCherry-DAT. 48 h later, cells were fixed and incubated with anti-HA and anti-mCherry primary antibodies followed by Alexa Fluor-labeled secondary antibodies. Images collected by confocal microscopy correspond to distribution of HA-GAT-1 variants (green channel), mCherry-DAT (red channel), or the merge. Scale bar: 10 µm.

Figure S2

Screenshot of the protein region around G307. **(A)**. In G307WT, a glycine located at the cytoplasmic end of TM6 makes polar and carbonyl and hydrogen bonds with adjacent residues (from L303 to Y309), as well as with G105 and Q106 in the cytoplasmic end of TM2. (**B**) In the mutant G307R, the lateral group of arginine has many novel interactions with TM2 (from E101 to I304) as well as with M487 located in the intracellular loop 5, IC5. Color code: clash, pink lines; hydrophobic, green lines; hydrogen bonds, red lines; carbonyl, dark blue; van der Waals, light blue. A detailed spin animation of this region is presented in supplementary video 1 (G307WT) and supplementary video 2 (G307R).

It can be appreciated that in G307WT, the peptide carboxyl and amino groups of glycine establish hydrogen bonds and polar interactions with the nearby residues L303, I304 and Y309, all in TM6b, and with G105 and Q106 in the cytoplasmic end of TM2. However, arginine, apart from these interactions, establishes a multitude of clash interactions through its lateral chain, especially with TM2. Moreover, charged amino groups from the R group are inserted into TM2 where they establish numerous additional polar interactions. Also, additional interactions are observed with N310 and M457 (IC5).

Figure S1

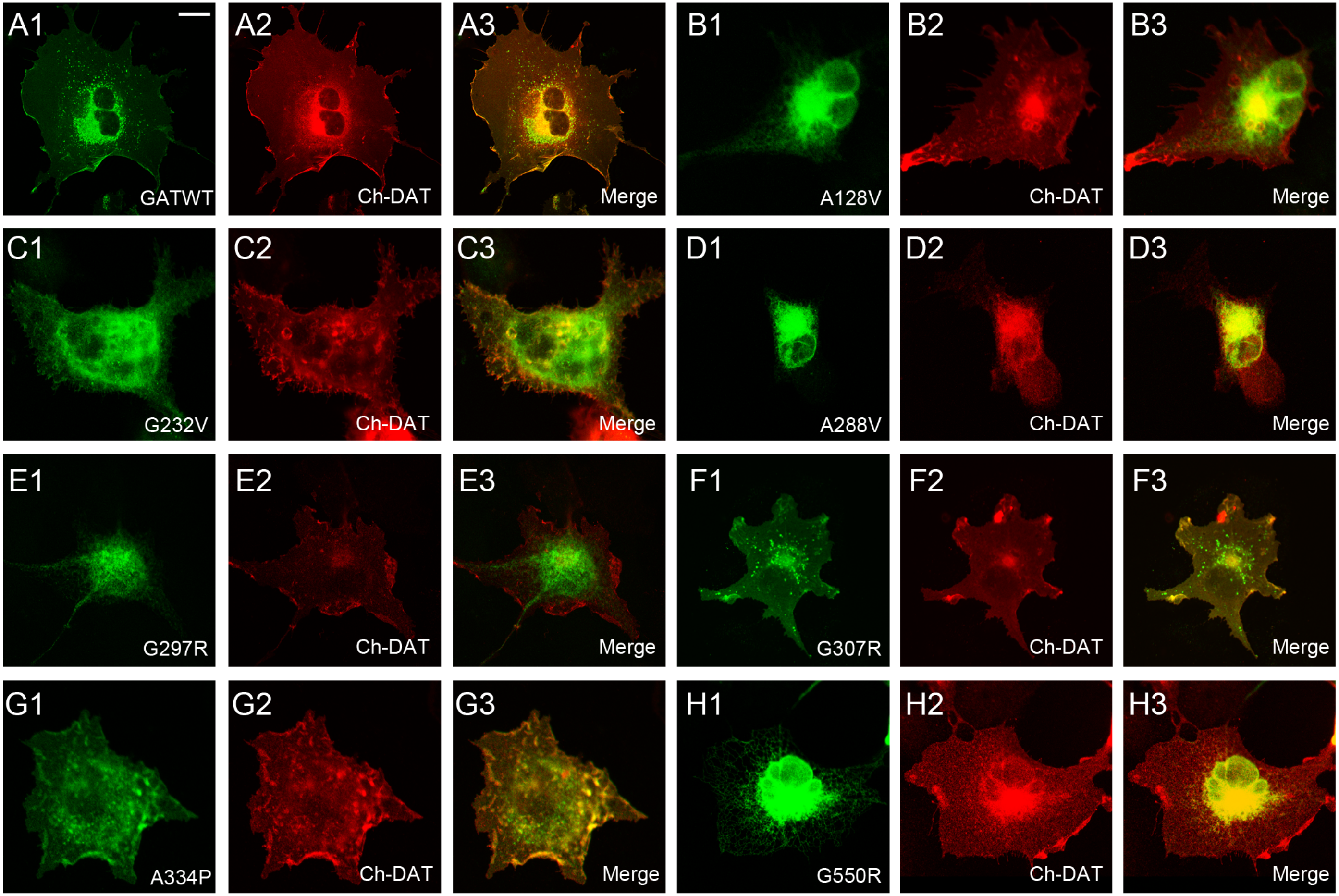
A1
GATWT
A2
Ch-DAT
A3
Merge
B1
A128V
B2
Ch-DAT
B3
Merge
C1
G232V
C2
Ch-DAT
C3
Merge
D1
A288V
D2
Ch-DAT
D3
Merge
E1
G297R
E2
Ch-DAT
E3
Merge
F1
G307R
F2
Ch-DAT
F3
Merge
G1
A334P
G2
Ch-DAT
G3
Merge
H1
G550R
H2
Ch-DAT
H3
Merge

Figure S2

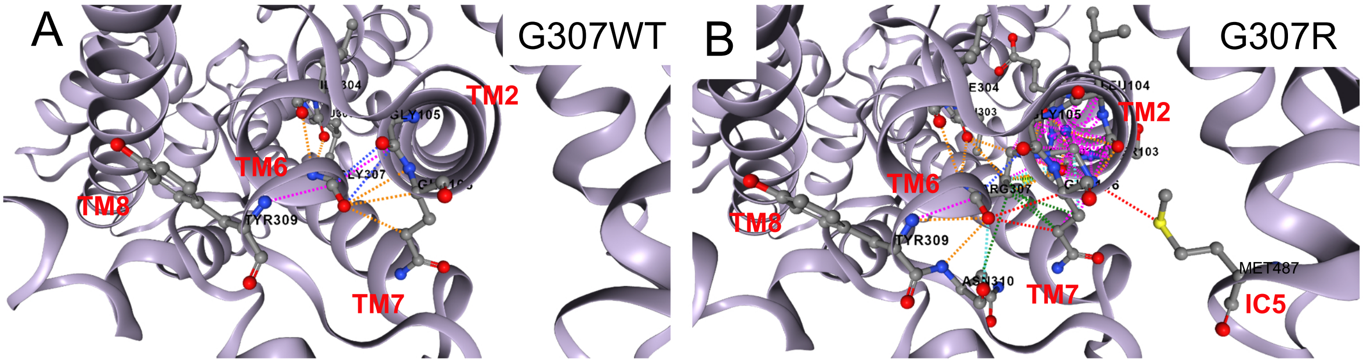
A
G307WT
TM2
TM6
TM8
GLY105
TYR309
TM7
B
G307R
LEU104
TM2
TM6
TM8
TYR309
MET487
TM7
IC5
